# Epidermal growth factor receptor tyrosine kinase inhibitors for de novo T790M mutation: A retrospective study of 44 patients

**DOI:** 10.1111/1759-7714.14272

**Published:** 2022-05-28

**Authors:** John Wen‐Cheng Chang, Chen‐Yang Huang, Yueh‐Fu Fang, Ching‐Fu Chang, Cheng‐Ta Yang, Chih‐Hsi Scott Kuo, Ping‐Chih Hsu, Chiao‐En Wu

**Affiliations:** ^1^ Division of Hematology‐Oncology, Department of Internal Medicine, Chang Gung Memorial Hospital at Linkou Chang Gung University College of Medicine Taoyuan Taiwan; ^2^ Division of Thoracic Medicine, Department of Internal Medicine, Chang Gung Memorial Hospital at Linkou Chang Gung University College of Medicine Taoyuan Taiwan

**Keywords:** afatinib, lung cancer, osimertinib, T790M mutation

## Abstract

**Background:**

This study aimed to evaluate possible treatment strategies for patients with de novo T790M mutation‐positive (T790M+) non‐small‐cell lung cancer (NSCLC).

**Methods:**

Patients diagnosed with de novo T790M+ NSCLC and treated with epidermal growth factor receptor tyrosine kinase inhibitors (EGFR‐TKIs) between 2011 and 2018 at a regional hospital in Taiwan were retrospectively reviewed. Their clinicopathological characteristics and subsequent treatment information were collected, and potential prognostic factors were identified using univariate and multivariate analyses.

**Results:**

All tumors with T790M mutations coexisted with sensitizing mutations. Through the last follow‐up in May 2021, afatinib and osimertinib demonstrated better progression‐free survival (PFS, *p* < 0.01) and overall survival (OS, *p* < 0.01) than gefitinib and erlotinib. Additionally, patients with low T790M ratios had better PFS than those with high T790M ratios, implying that the proportion of T790M+ tumors determined the response to EGFR‐TKIs. Multivariate analysis confirmed that both EGFR‐TKI treatment (osimertinib hazard ratio [HR] 0.06, 95% confidence interval [CI] 0.01–0.30; afatinib HR 0.09, 95% CI 0.02–0.39) and a low T790M ratio (HR 0.29, 95% CI 0.12–0.69) were independently favorable prognostic factors for patients with de novo T790M+ NSCLC. Median PFS was 6.1 (95% CI 4.4–7.8) months. In addition, patients treated with first‐generation (1G)/second‐generation (2G) EGFR‐TKIs followed by osimertinib (*n* = 8) demonstrated the best OS compared with patients treated with frontline osimertinib (*n* = 5) or 1G/2G EGFR‐TKIs without osimertinib (*n* = 28, *p* < 0.01).

**Conclusion:**

Sequential TKIs may represent an alternative option for de novo T790M mutation, particularly frontline afatinib and tumors with low T790M ratios.

## Graphical abstract

Patients with high T790M ratios had better progression‐free survival than patients with low T790M ratios. Osimertinib, afatinib, and low T790M ratios were favorable prognostic factors. Patients with a particularly low de novo T790M mutation ratio should be treated by afatinib followed by osimertinib rather than frontline osimertinib. A subclone of T790M mutation tumors may influence the response to EGFR‐TKIs.

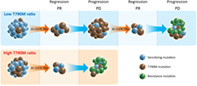

## INTRODUCTION

Molecular targeting using epidermal growth factor receptor tyrosine kinase inhibitors (EGFR‐TKIs), including first‐generation (1G) TKIs, such as gefitinib and erlotinib,^1^, ^2^, ^3^, ^4^ second‐generation (2G) TKIs, such as afatinib and dacomitinib,^5^, ^6^, ^7^, ^8^ and the third‐generation (3G) TKI, osimertinib,^9^, ^10^ have become the first‐line treatment for patients with advanced non‐small‐cell lung cancer (NSCLC) who harbor activating *EGFR* mutations (EGFRm+).^11^, ^12^ In patients with T790M mutation, the amino acid at position 790, typically a threonine residue, is replaced by a methionine, thereby reducing the binding affinity of TKI to EGFR.^13^ The acquired T790M mutation represents a major resistance mechanism that develops in more than half of NSCLC patients undergoing treatment with 1G/2G EGFR‐TKIs.^14^ Osimertinib was developed to overcome the T790M resistance mutation and is selective for both EGFR‐TKI sensitizing mutations and T790M resistance mutations, with demonstrated activity in patients who acquired T790M mutations following previous EGFR‐TKI treatment.^15^

In contrast to the acquired T790M mutation, de novo T790M mutations are found in treatment‐naïve NSCLC, and the frequency of de novo T790M mutations depends on the sensitivity of the methods used for mutation detection.^16^, ^17^, ^18^, ^19^ Osimertinib may be considered the first‐line choice for treatment in patients with a de novo T790M mutation. In the AURA study, which examined the effects of first‐line osimertinib treatment in EGFRm+ NSCLC, six of the seven patients (harboring the L858R mutation) with de novo T790M demonstrated a partial response (PR), the duration of response ranging from 6.9 to 27.7 months.^20^ However, in the IPASS study, five patients with de novo T790M mutation were treated with the 1G TKI gefitinib. Of them, three (60%) (harboring L858R) achieved PR, one (20%) achieved stable disease (SD, harboring the exon 19 deletion), and one (20%) experienced progressive disease (PD, no additional mutation), implying that a compound T790M mutation might be sensitive to EGFR‐TKIs other than osimertinib.^21^ Although previous studies showed that the presence of a de novo T790M mutation was associated with worse progression‐free survival (PFS) and overall survival (OS) in patients with advanced EGFRm+ NSCLC treated with 1G^16^, ^22^, ^23^, ^24^ and 2G^25^, ^26^ EGFR‐TKIs, most of these studies were performed prior to the development of osimertinib. Osimertinib treatment may be associated with better PFS than 1G/2G EGFR‐TKIs but may not be advantageous over 1G/2G EGFR‐TKIs in terms of OS due to the ability to use osimertinib in patients who progress following 1G/2G EGFR‐TKI treatment.

Previous studies have demonstrated conflicting results, and no direct comparison has been performed among EGFR‐TKIs due to the low prevalence of the de novo T790M mutation. Therefore, we aimed to compare the efficacy of different EGFR‐TKIs in de novo T790M mutation‐positive (T790M+) NSCLC patients to identify an alternative therapeutic strategy for the de novo T790M mutation.

## METHODS

### Patients and data collection

Patient data were obtained from the Cancer Registry System using the Chang Gung Research Database,^27^ an integrated and comprehensive database comprising multi‐institutional, standardized, and electronic medical records from all Chang Gung Memorial Hospitals (CGMHs) in Taiwan. A total of 44 de novo T790M+ NSCLC patients were identified who received EGFR‐TKI treatment from January 2011 to January 2018. Patients treated with concurrent chemotherapy, concurrent bevacizumab, and second‐line systemic or neoadjuvant treatments were excluded. The *EGFR* mutation status was retrospectively reviewed.

The clinical data of 44 patients who received EGFR‐TKIs as the first‐line treatment were retrospectively reviewed. Furthermore, the clinicopathological features, including age, sex, smoking history, Eastern Cooperative Oncology Group performance status (ECOG PS) score, tumor involvement, *EGFR* mutation, such as concurrent exon 19 deletion, L858R, or uncommon mutation, tumor response, and subsequent treatment, were obtained. The last follow‐up time point collected in this study was May 2021.

This study was approved by the Institutional Review Board of CGMH (201901395B0). Patient consent to participate was not required due to the retrospective nature of this study.

### *EGFR* mutation detection

Sanger sequencing was the only method for detecting *EGFR* mutation prior to 2012. The development of the amplification refractory mutation system (ARMS) increased the sensitivity of *EGFR* mutation detection, and after 2012 most patients' *EGFR* mutations have been detected using ARMS, such as QIAGEN and TaqMan assays unless the physicians required other methods. Using ARMS, delta CT (dCT) values could be obtained for T790M and other sensitizing mutations. Differences in the dCT values for the T790M mutation and sensitizing mutations (i.e. dCT [T790M] − dCT [sensitizing mutation]) indicated the ratio between the T790M mutation and other *EGFR* sensitizing mutations (T790M ratio) within the tumor sample. For more than two sensitizing mutations detected within the same tumor, the mutation with lower dCT was used as the reference. Due to the relatively low sensitivity of Sanger sequencing, any T790M mutation detected by Sanger sequencing was categorized as having a high T790M ratio in the present study. The present study evaluated the prognostic value of the T790M ratio for estimating PFS. The T790M ratio was found to be relatively variable, therefore it was classified into two subgroups, high and low T790M ratio, according to the median value.

### Treatment and response evaluation

All patients were administered EGFR‐TKIs once daily until disease progression or intolerable toxicity. The tumor response was evaluated using the Response Evaluation Criteria in Solid Tumors 1.1 criteria. The best clinical tumor response was recorded based on the radiological findings: the complete response (CR), PR, SD, or progressive disease (PD). Any tumor response that was not assessed before death or discontinuation due to intolerance was recorded as not assessed (NA). The objective response rate (ORR) was the sum of CR and PR, and the disease control rate (DCR) was the sum of CR, PR, and SD. In the survival analysis, PFS was defined as the duration from the first day of EGFR‐TKI treatment until the first radiological evidence of disease progression, EGFR‐TKI discontinuance due to toxicity or other reasons, death, or the latest follow‐up time point. Those patients who experienced no progression or death during the treatment were censored during PFS analysis. Patients with radiological progression or death within 1 month after EGFR‐TKI discontinuation and who received no sequential treatment were counted as an event. OS was defined as the duration from the first day of EGFR‐TKI treatment until the date of death or the last follow‐up. Data of patients who did not experience death were censored during survival analysis.

### Statistical analyses

The PFS and OS were estimated using the Kaplan–Meier method and compared using the log‐rank test. Univariate analysis was performed to evaluate possible prognostic factors, including age, sex, staging, T790M ratio, ECOG PS, smoking history, and tumor involvement. Multivariate analysis was performed, including all variables with *p* < 0.1 on univariate analysis, to evaluate the independent prognostic factors. The results are presented as the hazard ratio (HR) and 95% confidence interval (CI) based on Cox regression analyses. IBM SPSS Statistics for Windows (Version 23.0, Armonk) was used to perform all statistical analyses, and *p* < 0.05 was considered significant. We used the R packages “survival” and “survminer” to plot the survival curves.

## RESULTS

### Patient characteristics

A total of 2420 patients with EGFRm+ NSCLC treated with frontline EGFR‐TKIs were reviewed. Among them, 2190 (90.5%) patients had a common mutation (exon 19 deletion or L858R mutations), 186 (7.7%) had an uncommon mutation other than T790M, and 44 (1.8%) had de novo T790M mutation. The de novo T790M mutations were detected using the AMRS (23 by QIAGEN, 15 by TaqMan) in 38 patients, the cobas® EGFR Mutation Test in one patient, Sanger sequencing in four patients, and an unknown assay in one.

A total of 44 patients with T790M mutation retained for this study had a mean age of 71 years, and 32 (72.7%) patients were women. All but one patient presented metastatic NSCLC (stage IV). Among this population, 21 (47.7%), six (13.6%), 12 (27.3%), and five (11.4%) patients were treated with gefitinib, erlotinib, afatinib, and osimertinib, respectively, as first‐line treatments. Patients who received osimertinib and erlotinib were more likely to have experienced metastasis to the brain (*p* = 0.032; Table 1) prior to treatment. The ORR and DCR were 31.8% and 52.3%, respectively. Afatinib (ORR 50.0%, DCR 83.3%) and osimertinib (ORR 60.0%, DCR 80.0%) had numerically higher ORR and DCR than gefitinib (ORR 19.0%, DCR 38%) and erlotinib (ORR 16.7%, DCR, 16.7%); however, statistical significance was not noted due to limited sample size.

**TABLE 1:** Baseline characteristics

Variables	All patients (*n* = 44)	Gefitinib (*n* = 21)	Erlotinib (*n* = 6)	Afatinib (*n* = 12)	Osimertinib (*n* = 5)	*p* value
Age (years), median (IQR)	71 (18)	70 (17)	74.5 (34)	68 (19)	75 (23)	0.788
<70	21 (47.7)	11 (52.4)	2 (33.3)	6 (50.0)	2 (40.0)	0.885
≥70	23 (52.3)	10 (47.6)	4 (66.7)	6 (50.0)	3 (60.0)	
Sex						0.847
Male	12 (27.3)	6 (28.6)	1 (16.7)	3 (25.0)	2 (40.0)	
Female	32 (72.7)	15 (71.4)	5 (83.3)	9 (75.0)	3 (60.0)	
ECOG PS						0.644
0–1	30 (68.2)	15 (71.4)	3 (50.0)	9 (75.0)	3 (60.0)	
2–4	14 (31.8)	6 (28.6)	3 (50.0)	3 (25.0)	2 (40.0)	
Smoking status						0.408
Ever	9 (20.5)	3 (14.3)	2 (33.3)	2 (16.7)	2 (40.0)	
Never	35 (79.5)	18 (85.7)	4 (66.7)	10 (83.3)	3 (60.0)	
Mutation type						0.563
Del19	7 (15.9)	3 (14.3)	1 (16.7)	2 (16.7)	1 (20.0)	
L858R	8 (18.2)	5 (23.8)	2 (33.3)	1 (8.3)	0	
L858R/Del19	24 (54.5)	12 (57.1)	3 (50.0)	7 (58.3)	2 (40.0)	
Others	5 (11.4)	1 (4.8)	0	2 (16.7)	2 (40.0)	
Metastatic sites						
Lung	21 (47.7)	10 (47.6)	4 (66.7)	5 (41.7)	2 (40.0)	0.813
Liver	4 (9.1)	2 (9.5)	1 (16.7)	0	1 (20.0)	0.300
Brain	10 (22.7)	3 (14.3)	3 (50.0)	1 (8.3)	3 (60.0)	0.032
Bone	21 (47.7)	10 (47.6)	4 (66.7)	5 (41.7)	2 (40.0)	0.813
Pleura	26 (59.1)	15 (71.4)	4 (66.7)	6 (50.0)	1 (20.0)	0.194
T790M ratio						0.082
≥Median	21 (47.7)	12 (57.1)	3 (50.0)	3 (25.0)	3 (60.0)	
<Median	20 (45.5)	8 (38.1)	1 (16.7)	9 (75.0)	2 (40.0)	
Unknown	3 (6.8)	1 (4.8)	2 (33.3)	0	0	
Response						0.128
PR	14 (31.8)	4 (19.0)	1 (16.7)	6 (50.0)	3 (60.0)	
SD	9 (20.5)	4 (19.0)	0	4 (33.3)	1 (20.0)	
PD	15 (34.1)	10 (47.6)	3 (50.0)	1 (8.3)	1 (20.0)	
NA	6 (13.6)	3 (14.3)	2 (33.3)	1 (8.3)	0	
Subsequential treatment						
Chemotherapy	20 (45.5)	12 (57.1)	2 (33.3)	4 (33.3)	2 (40.0)	0.559
Osimertinib	9 (20.5)	3 (14.3)	0	5 (41.7)	1 (20.0)	0.166
1G/2G TKIs	10 (22.7)	5 (23.8)	1 (16.7)	2 (16.7)	2 (40.0)	0.786
Bevacizumab	3 (6.8)	1 (4.8)	0	0	2 (40.0)	0.051
Immunotherapy	1 (2.3)	0	0	0	1 (20.0)	0.114

*Note*: Data are presented as the number (percentage) unless otherwise stated.

Abbreviations: 1G/2G TKIs, first‐generation/second‐generation tyrosine kinase inhibitors; ECOG PS, Eastern Cooperative Oncology Group performance status; IQR, interquartile range; NA, not assessed; PD, progressive disease; PR, partial response; SD, stable disease.

### Influence of TKIs and other variables on PFS

**FIGURE 1:**
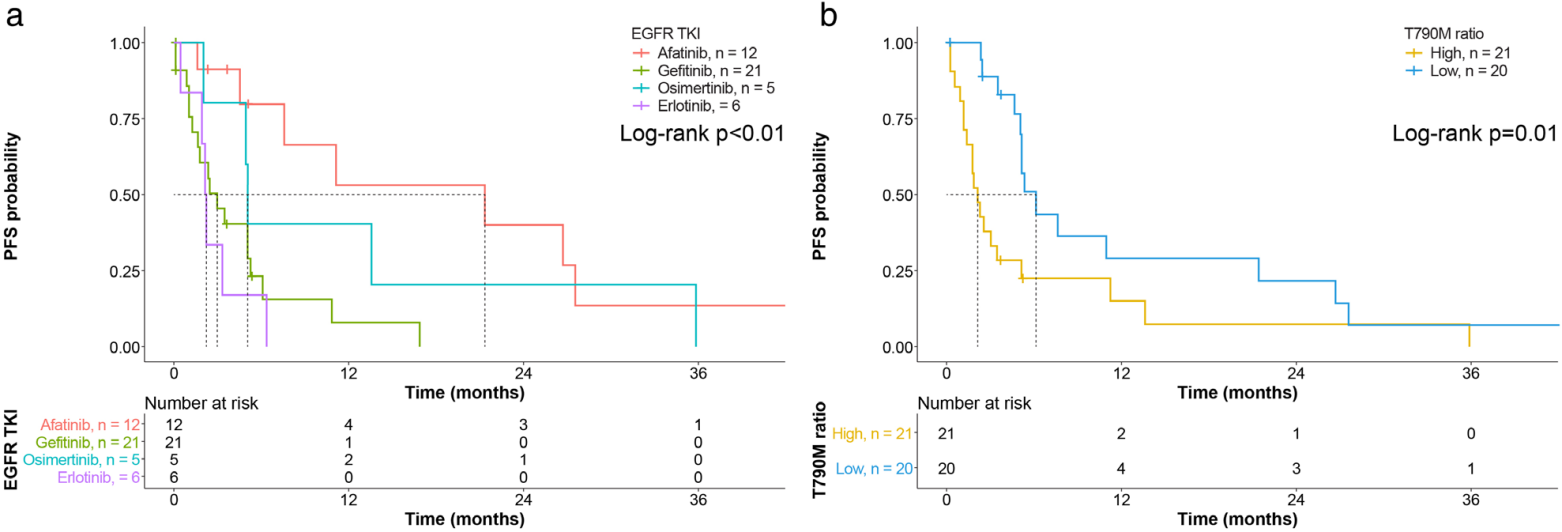
Kaplan–Meier curves showing progression‐free survival (PFS) for a group of 44 patients treated with different epidermal growth factor receptor tyrosine kinase inhibitors (EGFR‐TKIs) (a) and based on the T790M ratio (b). The survival curves were compared across the groups using the log‐rank test. The vertical dotted lines represent the median time to disease progression

On an individual basis, the TKI used and the T790M ratio were significantly associated with PFS (all *p* < 0.05). Patients who received afatinib (median PFS 21.4 months, 95% CI 3.5–39.3 months) and osimertinib (median PFS 5.1 months, 95% CI 4.8–5.5 months) had significantly longer PFS than patients who received erlotinib (median PFS 2.2 months, 95% CI 1.9–2.5 months, *p* = 0.002; Figure 1a). Furthermore, patients whose T790M ratio was lower than the median (low T790M ratio, median PFS 6.1 months, 95% CI 4.4–7.8 months) were less likely to experience disease progression than those with a high T790M ratio (median PFS 2.1 months, 95% CI 1.3–2.8 months, *p* = 0.037; Figure 1b).

On multivariate regression, after enrolling factors with *p* value <0.1 according to the univariate analysis, TKI, T790M ratio, and liver metastasis were independently associated with PFS. Patients who received afatinib (adjusted HR [AHR] 0.09, 95% CI 0.02–0.39, *p* = 0.001) and osimertinib (AHR 0.06, 95% CI 0.01–0.30, *p* < 0.001) were significantly associated with lower risks of disease progression relative to the patients who received erlotinib. Similarly, patients with a low T790M ratio were less likely to experience disease progression than those with a high T790M ratio (AHR 0.29, 95% CI 0.12–0.69, *p* = 0.005). Moreover, patients with no liver metastasis had a reduced risk of disease progression compared with those who experienced liver metastasis (AHR 0.21, 95% CI 0.06–0.69, *p* = 0.010; Table 2).

**TABLE 2:** Univariate and multivariate analysis of progression‐free survival

Variables	Total no.	Univariate			Multivariate	
		Median (months)	95% CI of median	*p* value	HR (95% CI)	*p* value
Age (years)				0.976		
<70	21	5.09	1.16–9.03			
≥70	23	4.60	2.06–7.14			
Sex				0.531		
Male	12	5.0	2.2–7.7			
Female	32	4.6	1.4–7.8			
ECOG PS				0.524		
0–1	30	5.0.	2.3–7.6			
2–4	14	3.5	0.1–7.2			
Smoking status				0.175		
Ever	9	5.0	1.4–8.5			
Never	35	5.1	2.3–7.8			
Mutation type				0.102		
Del19	7	6.1	3.2–8.9			
L858R	8	1.7	0.5–2.9			
L858R/Del19	24	5.1	2.1–8.1			
Others	5	5.1	0.1–15.2			
Lung metastasis				0.206		
Yes	21	4.6	0.8–8.4			
No	23	5.3	1.8–8.8			
Liver metastasis				0.074		
Yes	4	2.0	0.1–4.4		Reference	
No	40	5.1	3.0–7.2		0.21 (0.06–0.69)	0.010
Brain metastasis				0.379		
Yes	10	6.1	4.1–8.1			
No	34	3.5	0.9–6.1			
Bone metastasis				0.848		
Yes	21	4.6	2.0–7.2			
No	23	5.1	1.3–8.9			
Pleural metastasis				0.100		
Yes	26	2.5	0.1–5.4			
No	18	5.1	0.1–14.5			
T790M ratio				0.037		
≥Median	21	2.1	1.3–2.8		Reference	
<Median	20	6.1	4.4–7.8		0.29 (0.12–0.69)	0.005
Unknown	3	6.4	0.1–13.5		0.11 (0.03–0.48)	0.003
TKI				0.002		
Gefitinib	21	3.0	1.3–4.6		0.52 (0.19–1.47)	0.216
Erlotinib	6	2.2	1.9–2.5		Reference	
Afatinib	12	21.4	3.5–39.3		0.09 (0.02–0.39)	0.001
Osimertinib	5	5.1	4.8–5.5		0.06 (0.01–0.30)	<0.001
Response				<0.0001	–	
PR	14	7.6	0.1–15.7			
SD	9	11.2	2.2–20.3			
PD	15	2.1	1.4–2.7			
NA	6	1.2	0.1–2.3			

Abbreviations: CI, confidence interval; ECOG PS, Eastern Cooperative Oncology Group performance status; HR, hazard ratio; NA, not assessed; PD, progressive disease; PR, partial response; SD, stable disease; TKI, tyrosine kinase inhibitor.

### Influence of TKIs and other variables on OS

**FIGURE 2:**
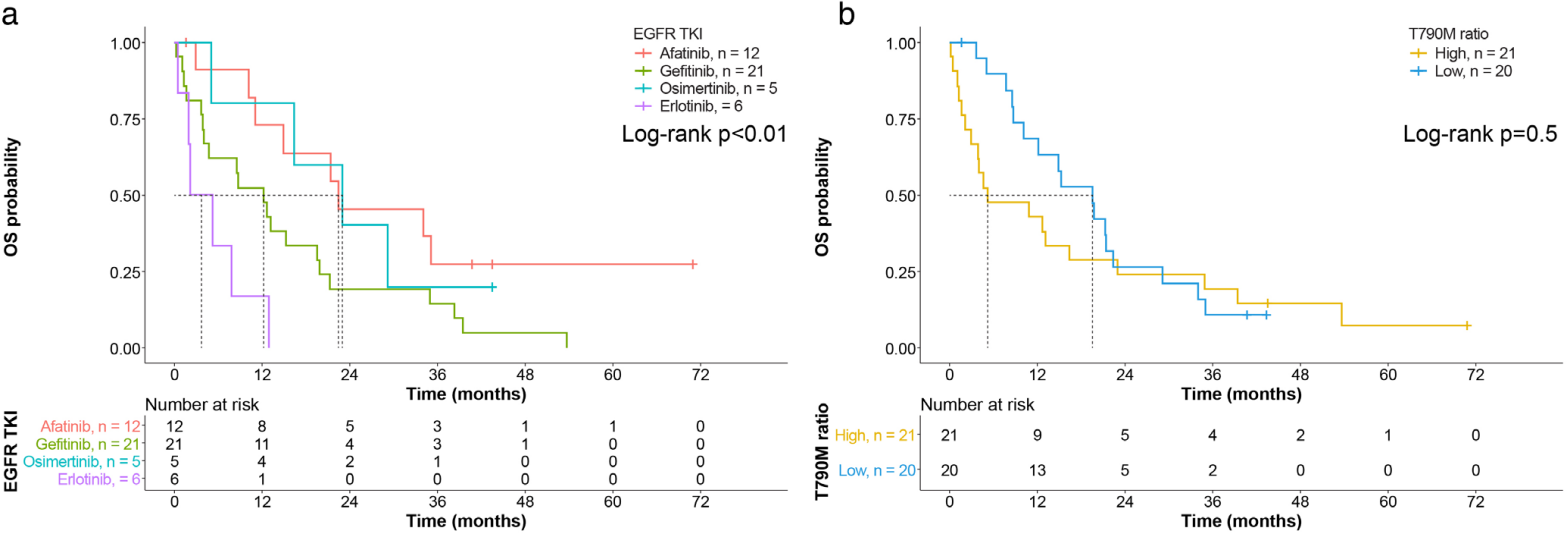
Kaplan–Meier curves showing overall survival (OS) for a group of 41 patients treated with epidermal growth factor receptor tyrosine kinase inhibitors (EGFR‐TKIs) (a) and based on the T790M ratio (b). The survival curves were compared across the groups using the log‐rank test. The vertical dotted lines represent the median time to death

TKI, sex, smoking status, and ECOG PS were individually associated with OS in the univariate regression analysis. Patients treated with afatinib (median OS 22.4 months, 95% CI 1.7–43.2 months), osimertinib (median OS 23.0 months, 95% CI 8.9–37.1 months), and gefitinib (median OS 12.2 months, 95% CI 6.0–18.3 months) were significantly associated with a lower risk of death compared with those treated with erlotinib (median OS 2.2 months, 95% CI 0.1–6.0 months, *p* = 0.002) (Figure 2a). Male sex, smoking, and ECOG PS of 2–4 were associated with a higher risk of death (all *p* < 0.05). Unlike the contribution of the T790M ratio to PFS, the T790M ratio had no impact on OS (Figure 2b; log‐rank *p* = 0.5).

Including all factors with a *p* value <0.1 according to the univariate analysis, TKI, ECOG PS, and lung metastasis were independently associated with OS after adjusting for smoking status and sex in the multivariate regression analysis. Patients treated with afatinib (AHR 0.23, 95% CI 0.07–0.81, *p* = 0.022) and osimertinib (AHR 0.21, 95% CI 0.06–0.8, *p* = 0.025) were significantly associated with lower risks of death, whereas those with lung metastasis (AHR 0.42, 95% CI 0.19–0.92, *p* = 0.029) and ECOG PS of 2–4 (AHR 0.32, 95% CI 0.14–0.74, *p* = 0.007) were significantly associated with a higher risk of death after adjusting for potential confounders (Table 3).

**TABLE 3:** Univariate and multivariate analyses of overall survival

Variables	Total no.	Univariate			Multivariate	
		Median (months)	95% CI of median	*p* value	HR (95% CI)	*p* value
Age (years)				0.510		
<70	21	12.9	9.7–16.1			
≥70	23	14.9	4.4–25.4			
Sex				0.042		
Male	12	5.2	0.1–13.8		0.45 (0.12–1.67)	0.233
Female	32	14.9	5.8–24.0		Reference	
ECOG PS				0.002		
0–1	30	19.8	9.3–30.4		0.32 (0.14–0.74)	0.007
2–4	14	7.8	0.1–18.5		Reference	
Smoking status				0.049		
Ever	9	5.2	4.9–5.5		Reference	
Never	35	15.3	10.3–20.3		0.61 (0.15–2.43)	0.483
Mutation type				0.281		
Del19	7	22.4	15.1–29.8			
L858R	8	2.9	0.4–5.3			
L858R/Del19	24	12.9	8.2–17.6			
Others	5	5.1	0.1–17.3			
Lung metastasis				0.052		
Yes	21	12.7	6.7–18.8		Reference	
No	23	15.3	3.2–27.3		0.42 (0.19–0.92)	0.029
Liver metastasis				0.105		
Yes	4	2.0	0.1–4.5			
No	40	13.2	9.4–17.0			
Brain metastasis				0.642		
Yes	10	12.9	8.0–17.7			
No	34	12.7	5.8–19.6			
Bone metastasis				0.475		
Yes	21	14.9	7.1–22.7			
No	23	12.7	8.0–17.4			
Pleural metastasis				0.242		
Yes	26	12.7	8.9–16.5			
No	18	15.3	4.9–25.6			
T790M ratio				0.773		
≥Median	21	5.2	0.1–15.6			
<Median	20	19.6	12.5–26.6			
Unknown	3	12.9	0.1–30.2			
TKI						
Gefitinib	21	12.2	6.0–18.3	0.002	0.61 (0.22–1.74)	0.355
Erlotinib	6	2.2	0.1–6.0		Reference	
Afatinib	12	22.4	1.7–43.2		0.23 (0.07–0.81)	0.022
Osimertinib	5	23.0	8.9–37.1		0.21 (0.06–0.83)	0.025
Response				<0.0001	**–**	
PR	14	22.4	8.0–36.8			
SD	9	16.4	13.1–19.7			
PD	15	4.7	3.1–6.3			
NA	6	1.2	0.1–2.7			

Abbreviations: CI, confidence interval; ECOG PS, Eastern Cooperative Oncology Group performance status; HR, hazard ratio; NA, not assessed; PD, progressive disease; PR, partial response; SD, stable disease; TKI, tyrosine kinase inhibitor.

### Subsequent osimertinib after first‐line treatment

Through the most recent follow‐up, only one patient treated with afatinib was found to remain on first‐line treatment, characterized as PR and progression‐free for 43.5 months. Osimertinib is the standard treatment for patients with acquired T790M, and eight patients received subsequent osimertinib, whereas 28 patients did not receive subsequent osimertinib. Two patients had unknown subsequent treatments due to loss to follow‐up during first‐line treatment.

Further analysis of OS was performed according to subsequent treatment. Patients who received subsequent osimertinib treatment following 1G/2G EGFR‐TKIs (median OS 37.3 months, 95% CI 35.0–not reached, NR) had longer OS than patients without subsequent osimertinib (median OS 23.0 months, 95% CI 16.4–NR) and patients treated with osimertinib alone (median OS 8.2 months, 95% CI 3.9–13.2, *p* < 0.001).

## DISCUSSION

In the present study, 44 patients with de novo T790M+ NSCLC treated with EGFR‐TKIs were retrospectively reviewed. Both afatinib and osimertinib demonstrated compatible clinical activities based on ORR, DCR, and PFS analyses. In addition, the T790M ratio was significantly associated with PFS in de novo T790M+ NSCLC patients treated with EGFR‐TKIs, implying that a subclone of T790M mutation tumors influenced the response to EGFR‐TKIs. Furthermore, patients who received sequential treatment (1G/2G EGFR‐TKIs followed by osimertinib) experienced better survival than patients who received frontline line osimertinib or 1G/2G EGFR‐TKIs without subsequent osimertinib treatment.

The rate of de novo T790M mutation detection depends on the sensitivity of the methods used for mutation detection.^16^, ^17^, ^18^, ^19^ A meta‐analysis by Chen et al.,^19^ including 15 observational studies and three randomized controlled trials, analyzed and classified all reported detection methods into three categories based on the detection limit. Low‐sensitivity methods with T790M detection limits ≥5%, such as direct sequencing/ARMS, present a pooled detection rate of 3.92%. In contrast, intermediate‐sensitivity methods with detection limits of T790M equal to 0.1%–5% present a pooled detection rate of 27.62%, while high‐sensitivity methods with detection limits of T790M < 0.1% display the pooled detection rate of 63.99%. These findings imply that more than half of all EGFRm+ NSCLC tumors are likely to harbor an extremely low proportion (<0.1%) of T790M mutations even before EGFR‐TKI treatment. Although previous studies have shown that de novo T790M mutation is associated with worse PFS and OS in patients with advanced EGFRm+ NSCLC treated with 1G EGFR‐TKIs,^22^, ^23^, ^28^ not all patients have exhibited no response to 1G EGFR‐TKIs. In a study including 95 patients from the EURTAC trial, T790M has been detected using a highly sensitive method based on laser microdissection and peptide‐nucleic acid‐clamping PCR in 65.26% of patients. Although patients with a concurrent de novo T790M mutation have significantly shorter PFS than those without the T790M mutation (9.7 vs. 15.8 months, *p* = 0.0185),^28^ the response rates are 47.1% versus 68.75% for patients with and without T790M mutation, respectively, suggesting that some patients with very low frequency of T790M mutations can benefit with erlotinib treatment.

Although osimertinib can target both T790M and EGFR‐sensitizing mutations, the activity of osimertinib against EGFR sensitizing mutations may not be as good as 1G/2G EGFR‐TKIs.^29^, ^30^ In 33 treatment‐naïve NSCLC patients treated with osimertinib, the T790M ratio assessed by droplet digital PCR (ddPCR) in tumors has been shown to correlate with the osimertinib response, and patients with higher T790M ratios have a longer treatment history than those with lower T790M ratios.^31^ In 111 NSCLC patients with acquired T790M mutation treated with osimertinib after progression on 1G/2G EGFR‐TKIs, the T790M ratio assessed by ddPCR has shown a significant correlation with the osimertinib response.^32^ A similar study reported that a high T790M ratio, as determined by next‐generation sequencing (NGS), is associated with better osimertinib outcomes than a low T790M ratio.^33^ These findings suggest that a high T790M ratio may represent a good prognostic factor for patients with either de novo or acquired T790M mutations, indicating that osimertinib may be more active for T790M mutations than EGFR‐sensitizing mutations, consistent with the findings from in vitro cell lines.^29^, ^30^

**FIGURE 3:**
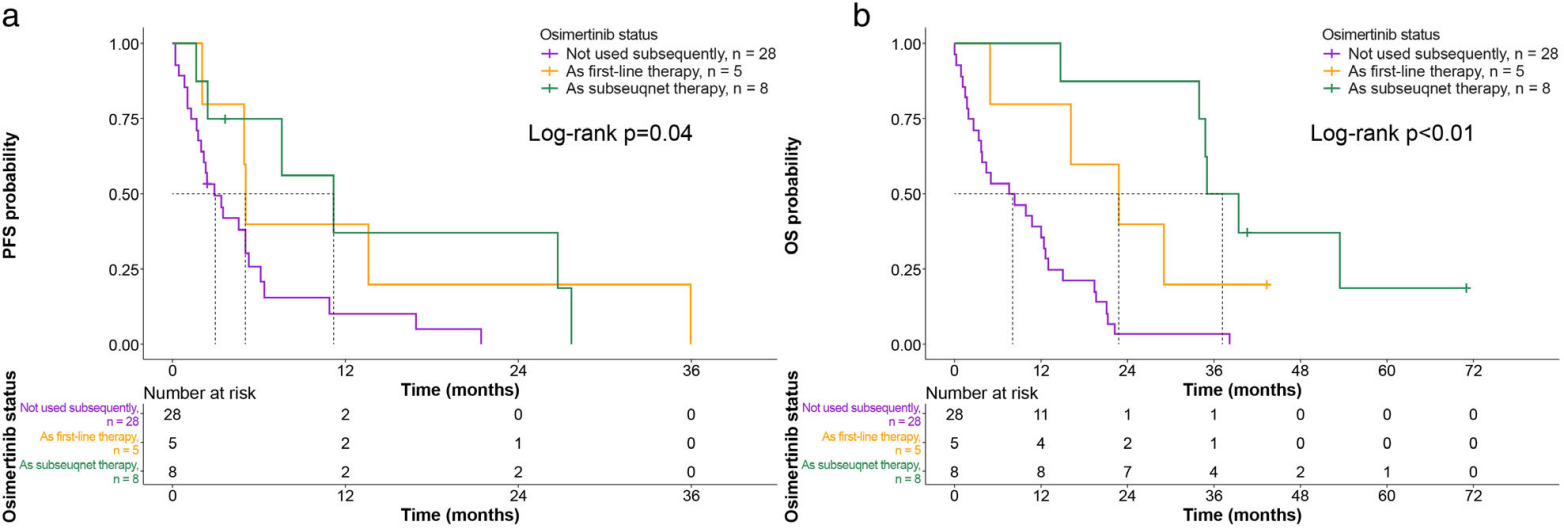
Kaplan–Meier curves showing progression‐free survival (PFS) (a) and overall survival (OS) (b) for a group of 41 patients based on the use of osimertinib as the first‐line treatment, as a subsequent treatment, or not at all. The survival curves were compared across the groups using the log‐rank test. The vertical dotted lines represent the median time to progression or death

Therefore, a current dilemma is whether frontline osimertinib or 1G/2G EGFR‐TKIs represent a better treatment for de novo T790M+ NSCLC patients. The latter treatment could present the opportunity for switching to osimertinib following progression on previous EGFR‐TKI treatment and lead to the best survival outcome, based on the results from our cohort (Figure 3). Theoretically, a T790M mutation should exist in tumors that progress after 1G/2G EGFR‐TKI treatment because of little TKI activity in the presence of the T790M mutation. Therefore, patients with a de novo T790M mutation, particularly patients with a low T790M ratio, should be treated using sequential TKIs (afatinib followed by osimertinib) rather than frontline osimertinib, based on the results of the present study (Figures 1 and 3).

This study has several limitations. First, this was a retrospective study enrolling limited cases, which also reflected the relative rarity of de novo T790M detection when using traditional detection methods in clinical practice. Second, limited cases treated with frontline osimertinib were included because osimertinib has only been available for the last few years and is not reimbursed by Taiwan national health insurance. Third, the T790M ratio was only a relative value estimated using ARMS, a semiquantitative method of detection; the T790M ratio evaluation using more sensitive methods, such as NGS or ddPCR (quantitative methods), is important and is warranted in the future. Fourth, only five and eight patients received osimertinib as frontline and subsequential treatment, respectively; hence, the survival benefit should be interpreted with caution. In addition, patients treated with frontline osimertinib tended to have brain metastasis, which might lead to worse OS than patients without brain metastasis. However, despite these limitations, this study was able to provide an alternative view based on the clinical findings when treating de novo T790M mutations with treatments other than osimertinib.

**FIGURE 4:**
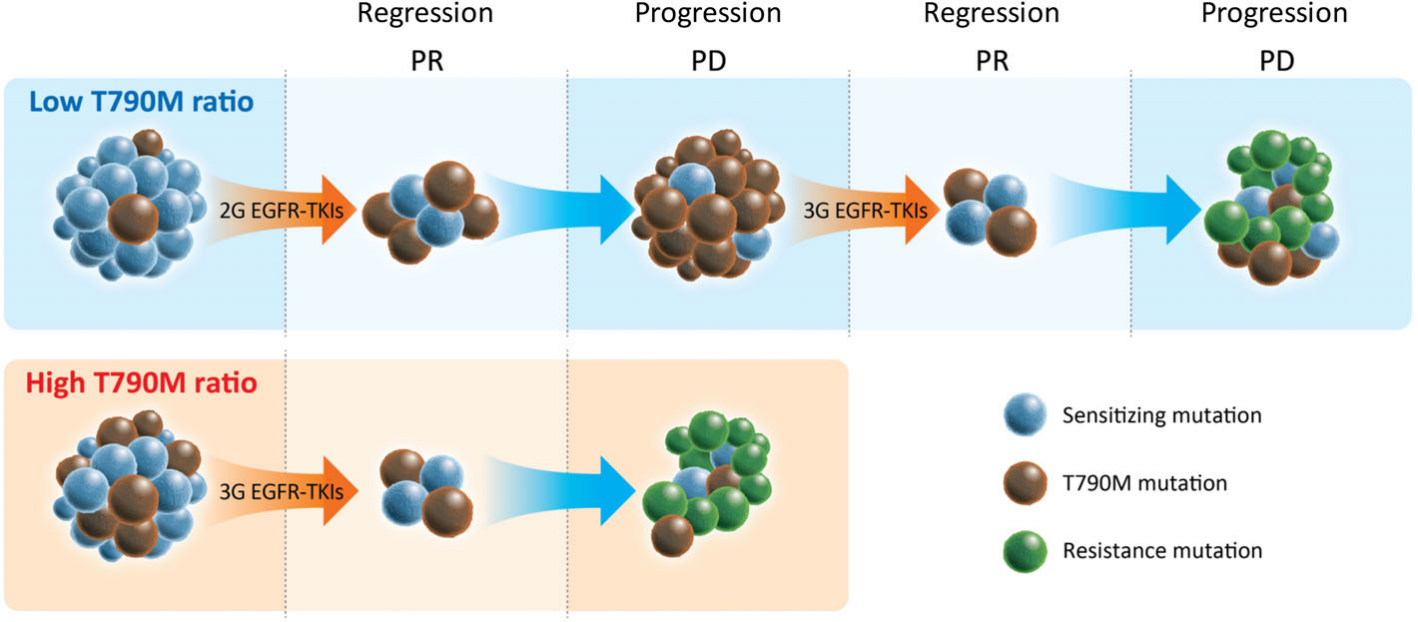
A proposed model of the treatment strategy for non‐small‐cell lung cancer harboring a de novo T790M mutation. For tumors with a low T790M ratio, treatment with second‐generation (2G) epidermal growth factor receptor tyrosine kinase inhibitors (EGFR‐TKIs) followed by third‐generation (3G) EGFR‐TKIs is recommended. In contrast, for tumors with a high T790M ratio, frontline 3G EGFR‐TKI treatment might be suitable

Our study provided evidence that sequential treatment with 1G/2G EGFR‐TKIs followed by osimertinib was effective in de novo T790M+ NSCLC patients. Afatinib was the best choice among the EGFK‐TKIs, particularly for patients with a low T790M ratio (Figure 4).

## CONFLICT OF INTEREST

The authors declare no conflicts of interest.
